# The application of additive manufacturing technology in pelvic surgery: A bibliometrics analysis

**DOI:** 10.3389/fbioe.2023.1123459

**Published:** 2023-04-06

**Authors:** Jian Li, Jiani Wang, Jia Lv, Junjun Bai, Shichao Meng, Jinxuan Li, Hua Wu

**Affiliations:** ^1^ Department of Orthopaedics, Third Hospital of Shanxi Medical University, Shanxi Bethune Hospital, Shanxi Academy of Medical Sciences, Tongji Shanxi Hospital, Taiyuan, China; ^2^ Department of Paediatric Medicine, Shanxi Medical University, Taiyuan, China; ^3^ Department of Orthopaedics, The Second Affiliated Hospital of Shanxi Medical University, Taiyuan, China; ^4^ Department of Orthopaedics, Tongji Hospital, Tongji Medical College, Huazhong University of Science and Technology, Wuhan, China

**Keywords:** pelvic surgery, three-dimensional printing technology, additive manufacturing technology, rapid prototyping, bibliometric analysis

## Abstract

With the development of material science, additive manufacturing technology has been employed for pelvic surgery, addressing the challenges, such as the complex structure of the pelvis, difficulty in exposing the operative area, and poor visibility, of the traditional pelvic surgery. However, only limited studies have been done to review the research hotspots and trends of the additive manufacturing technology applied for pelvic surgery. In this study, we comprehensively analyzed the literatures related to additive manufacturing technology in pelvic surgery by a bibliometrics analysis and found that additive manufacturing technology is widely used in several aspects of preoperative diagnosis, preoperative planning, intraoperative navigation, and personalized implants for pelvic surgery. Firstly, we searched and screened 856 publications from the Web of Science Core Collection (WoSCC) with TS = (3D printing OR 3D printed OR three-dimensional printing OR additive manufacturing OR rapid prototyping) AND TS = (pelvis OR sacrum OR ilium OR pubis OR ischium OR ischia OR acetabulum OR hip) as the search strategy. Then, 565 of these were eliminated by evaluating the titles and abstracts, leaving 291 pieces of research literature whose relevant information was visually displayed using VOSviewer. Furthermore, 10 publications with high citations were selected by reading all publications extensively for carefully evaluating their Titles, Purposes, Results, Limitations, Journal of affiliation, and Citations. Our results of bibliometric analysis demonstrated that additive manufacturing technology is increasingly applied in pelvic surgery, providing readers with a valuable reference for fully comprehending the research hotspots and trends in the application of additive manufacturing technology in pelvic surgery.

## Background

Additive manufacturing (AM), also referred to as three-dimensional (3D) printing technology, was invented in the 1980s and had been initially used for dentistry and maxillofacial surgery (Moss et al., 1991; Fuhrmann et al., 1996). Later, various types of additive manufacturing technology, such as 3D printing models, 3D virtual software, and 3D navigation systems, are developed and successfully applied in the medical field, particularly in pelvic surgery, including complex pelvic fractures (Wu et al., 2015), pelvic tumors (Wong et al., 2015), total hip arthroplasty (Arabnejad et al., 2017), and a variety of other pelvic surgeries (Wong et al., 2015; Upex et al., 2017). Additive manufacturing technology not only resolved the challenges of traditional pelvic surgery which occurred owing to the unique anatomical structure, limited visibility, and restricted working space of the pelvis (Coccolini et al., 2017), but also can be applied to preoperative planning, intraoperative navigation, and prosthetic plants, which can effectively improve surgical security and achieve individuation and accuracy in pelvic surgery (Wei et al., 2017). However, the progress and trends of the additive manufacturing technology in pelvic surgery were not systematically discussed by the bibliometrics analysis, limiting its wider application and inhibiting us from identifying the hotpots in pelvic surgery.

As a new discipline, bibliometrics is a good candidate for describing and analyzing the dynamics and progress of a specific research field (Peng et al., 2022). Bibliometrics analysis can comprehensively and systematically derive and visualize the detailed information (countries, institutions, authors, keywords, journals, references, and so on) of the published papers related to a specific research field. In addition, according to some scholars’ opinions (Zhao et al., 2022), visual co-citation analysis can help with data interpretation and make the results more comprehensive. Simultaneously, the internal relationships of these data can be extracted, allowing complex and difficult-to-understand data to be presented in the form of images. In addition, VOSviewer (Zhu et al., 2021), a research tool widely employed in the field of bibliometrics, offers three visual views, including network visualization, overlay visualization, and density visualization.

Therefore, with the help of the bibliometrics analysis, we may better understand the current research status and future research trends in a specific field, allowing us to better identify research hotspots. In this study, bibliometric was employed for systematic and specific analyzing the application of additive manufacturing technology in pelvic surgery. The purpose of this study is to describe the application status and future development trends of additive manufacturing technology in pelvic surgery by analyzing existing relevant literature in order to discover research hotspots in this field.

## Materials and methods

### Search strategies

The Web of Science Core Collection (WoSCC) (Yeung et al., 2021), served as the database for this paper, is widely regarded as the best database for bibliometrics. Specific literature retrieval strategies were shown in Table 1. A total of 856 documents were discovered using the established retrieval strategy. 565 of these were eliminated by reviewing the titles and abstracts, leaving 291 pieces of research literature. The original data from the selected literature was exported to a text format, from which we extracted the year of publication, title, language, abstract, journal, author, affiliation, document type, keywords, and citation count. Finally, we extensively read all publications and opted for the 10 highly cited publications for deep analysis, extracting relevant data such as author, title, journal, purpose, results, limitations, and citations.

**TABLE 1:** Summary of date source and selection.

category	Specific standard requirements
Research database	Web of science Core Collection
Citation indexes	SCI-EXPANDED
Searching period	January 2009 to November 2022
Language	“English”
Searching keywords	TS = (3D printing OR 3D printed OR three-dimensional printing OR additive manufacturing OR rapid prototyping) AND TS = (pelvis OR sacrum OR ilium OR pubis OR ischium OR ischia OR acetabulum OR hip)
Document types	“Articles”
Date extraction	Export with full records and cited references in plain text format
Sample size	856

### Data processing

VOSviewer (version 1.6.18) was employed to analyze the data for keywords, country, year of publication, institution, and journal, which were visually presented. Furthermore, read all publications extensively and choose highly 10 cited publications for detailed analysis and discussion.

## Results

### Publication outputs

**FIGURE 1:**
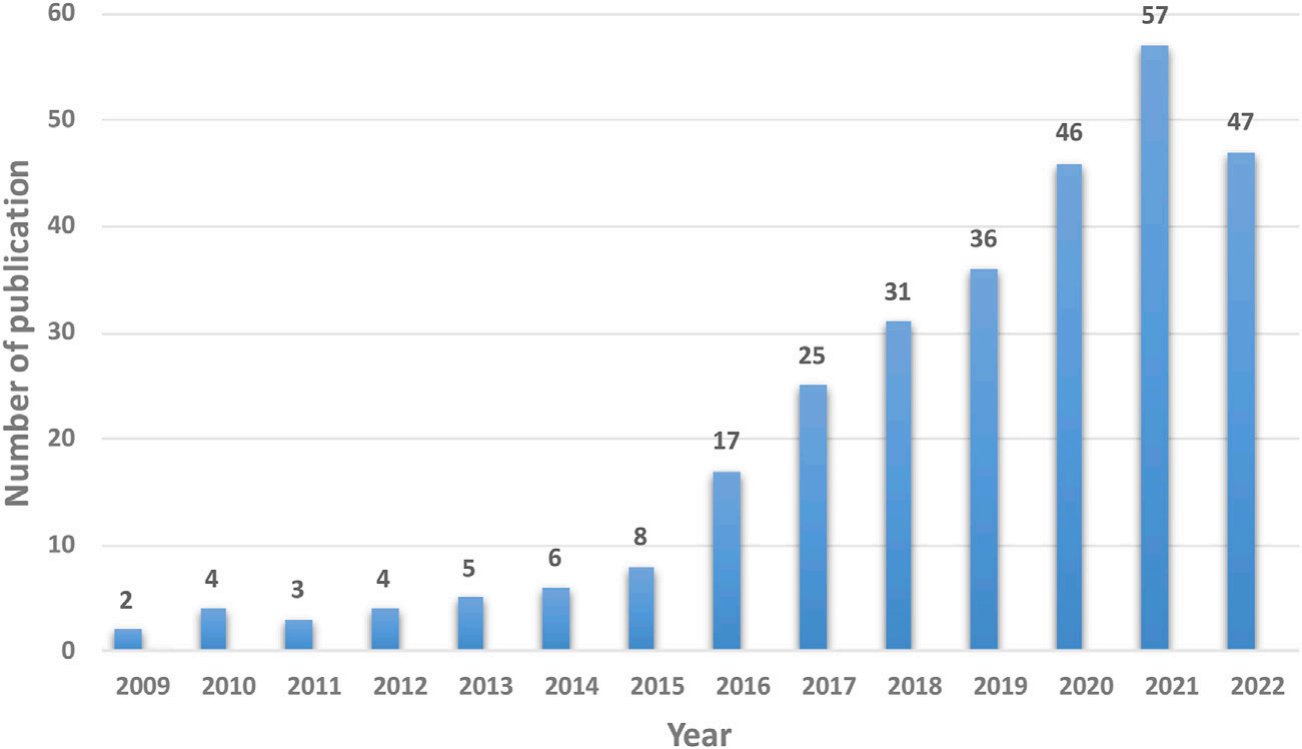
The annual trends of publications.

All 291 articles were published from January 2009 to November 2022 in 148 journals, written by 1,506 authors from 452 institutions in 45 countries, and cited 6,256 times by 1,835 different journals. During this period, the overall trends of research on the use of additive manufacturing technology in pelvic surgery increased from 2 articles in 2009 to 47 articles in 2022. The number of papers published between 1999 and 2015 was sporadic and modest. However, this number has rapidly increased since 2016, with the highest number of papers (57) published in 2021. The details were shown in Figure 1.

### Analysis of countries

**FIGURE 2:**
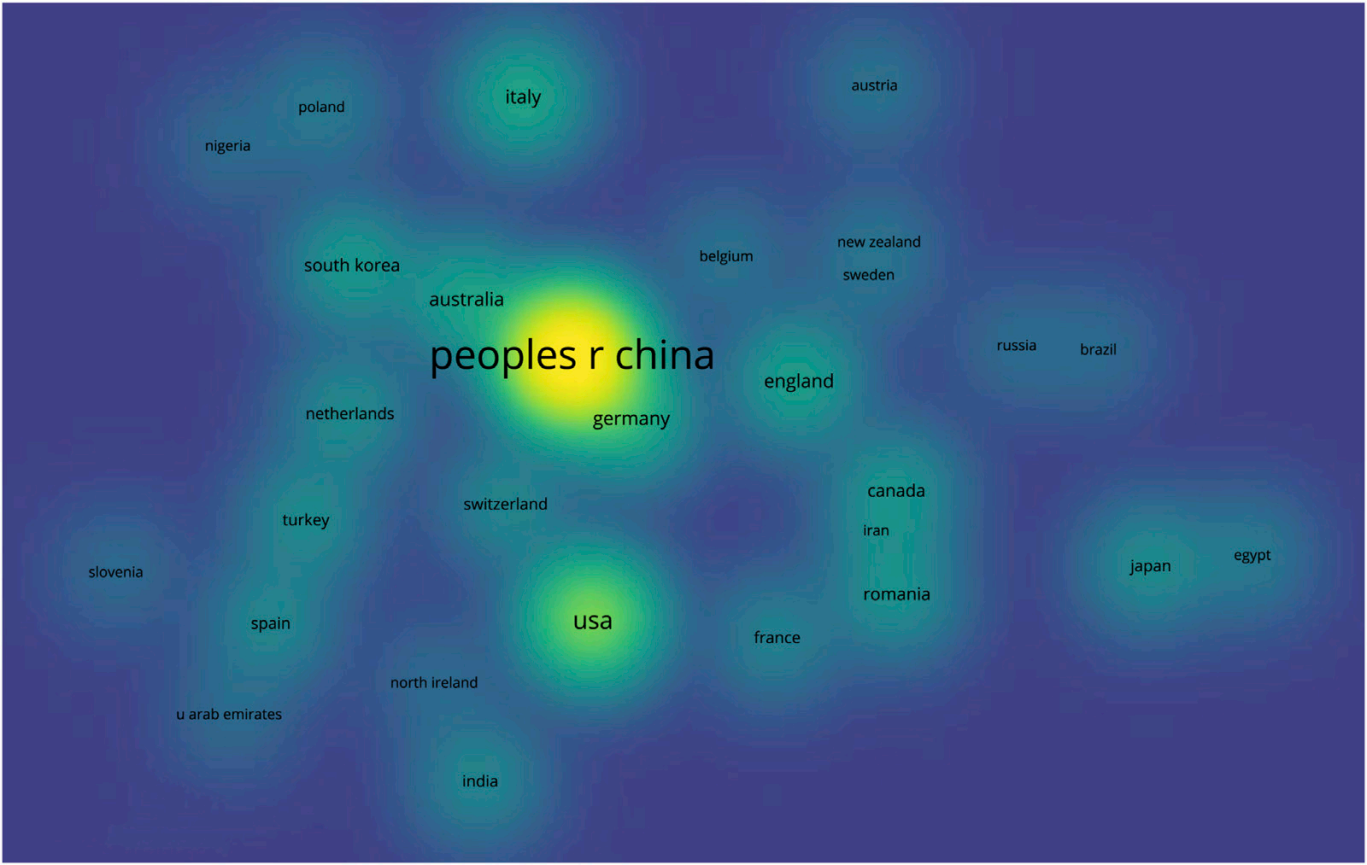
Density visualization of the distribution of all publications across countries. (Larger and brighter bubbles indicate the greatest number of publications in the field of additive manufacturing technology in pelvic surgery from a specific country, while closer bubbles in space indicate closer national collaborations.).

The selected articles on additive manufacturing technology in pelvic surgery were published in 54 countries. 41.2% (120/291) articles were published by China, followed by the United States (12.7%; 37/291), Germany (4.8%; 14/291), Italy (4.8%; 14/291), and Australia (4.1%; 12/291) (Table 2). Literature, published by China, had the most participants from other countries, indicating that they have more cooperation with other countries (Figure 2). In addition, as shown in Table 2, literature, published by China, had the highest average citation of 12.2, followed by that of the United States at 10.8, Australia at 9.6, Germany at 9.5, and Italy at 6.9.

**TABLE 2:** Top 5 countries contributed to research publications in the field of additive manufacturing technology in pelvic surgery.

Rank	Country	Publications	Percentage	Average citation
1	China	120	41.2	12.2
2	United State	37	12.7	10.8
3	Germany	14	4.8	9.5
4	Italy	14	4.8	6.9
5	Australia	12	4.1	9.6

### Analysis of institutions

**FIGURE 3:**
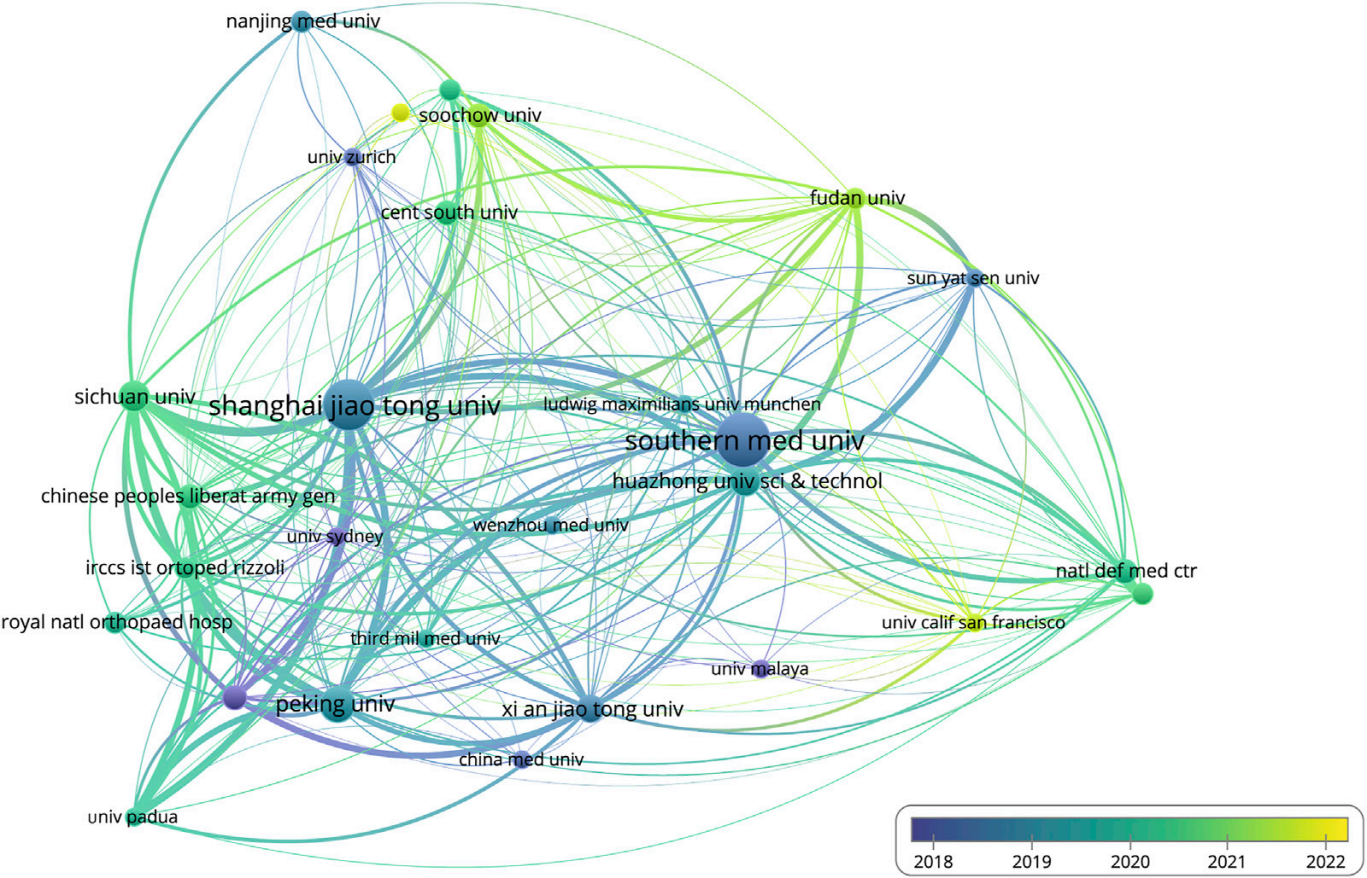
Visualization of institutional cooperation and publication period. (The size of the bubble represents the amount of paper issued by the institutions. The larger the bubble, the more paper is issued. In terms of time, the brighter the bubbles, the more recent research the organization has completed. Furthermore, the more connections there are between different bubbles, the closer the collaboration between different institutions will become.).

In terms of institutions, Southern Medical University published the highest number of articles (17, 5.8%), followed by Shanghai Jiao Tong University (16, 5.4%), Peking University (9, 3.1%), Huazhong University of Science and Technology (7, 2.4%), and Sichuan University (7, 2.4%). All top 5 institutions are from China (Table 3). The differences in publication volume were represented by the size of the bubbles, as shown in Figure 3, while the difference in publication time was symbolized by the color of the bubbles, with brighter colors indicating the most recent publications. More wires connecting bubbles means more collaboration between institutions. The top 5 institutions’ average citation was analyzed and shown in Table 3. Literature, published by Peking University, had the highest average citation of 28.1, followed by that of Shanghai Jiao Tong University at 16.1, Southern Medical University at 15, Huazhong University of Science and Technology at 11.3, and Sichuan University at 4.

**TABLE 3:** The top 5 most productive institutions in the field of additive manufacturing technology in pelvic surgery.

Rank	institutions	Publications	Percentage	Average citation
1	Southern Medical University	17	5.8	15
2	Shanghai Jiao Tong University	16	5.4	16.1
3	Peking University	9	3.1	28.1
4	Huazhong University of Science and Technology	7	2.4	11.3
5	Sichuan University	7	2.4	4

### Analysis of journals

**FIGURE 4:**
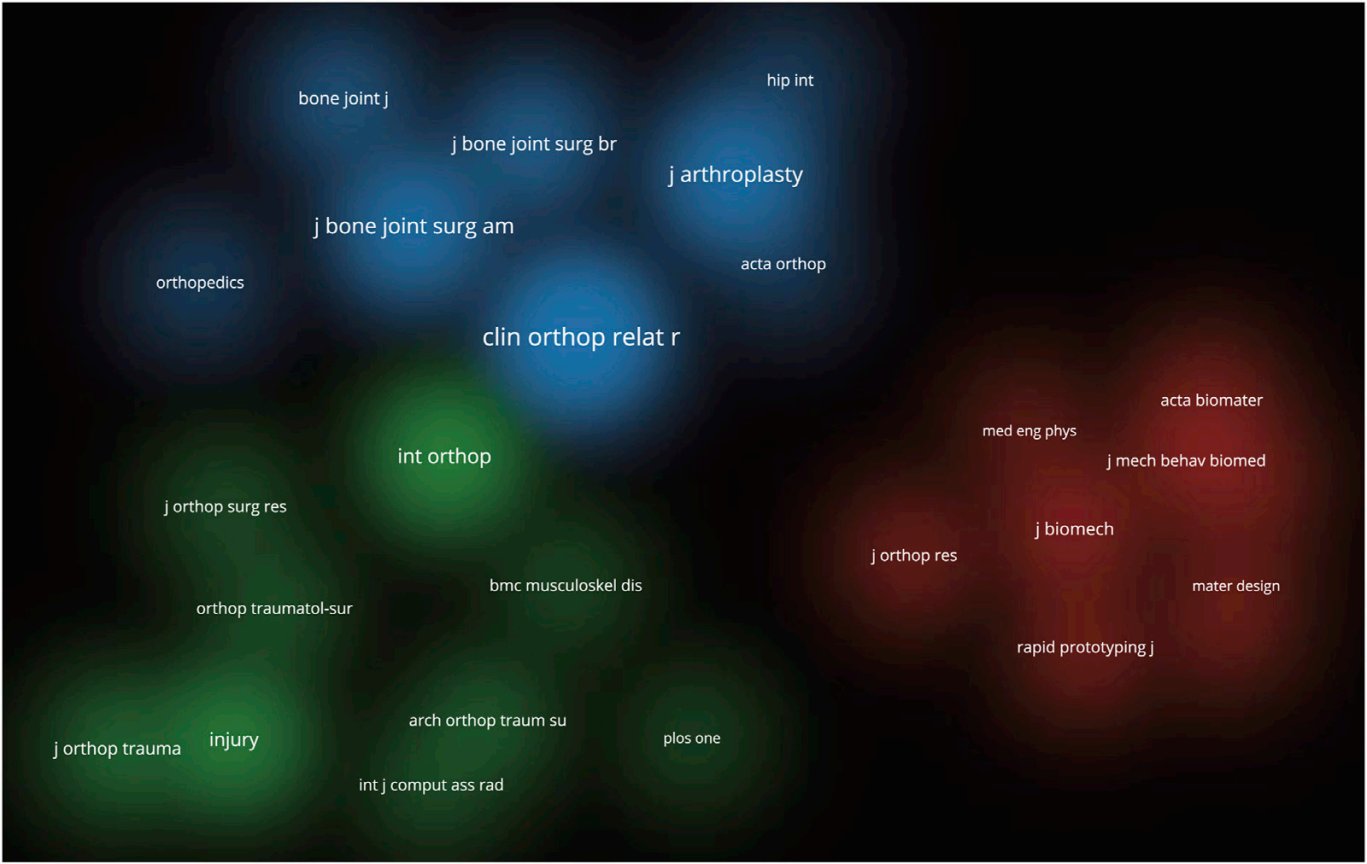
Density visualization of the co-cited journals. (A wider range of circles indicates that the journal has been cited more frequently. Different colors represent different clusters, so the co-cited journals can be seen to be divided into three clusters.).

The 291 articles on additive manufacturing technology in pelvic surgery published in different journals. International Orthopaedics has published the most literature (15 articles, 10.1%), followed by the Journal of Orthopaedic Surgery and Research (10 articles, 6.8%), Orthopaedic Surgery (9 articles, 6.1%), BMC Musculoskeletal Disorders (8 articles, 5.4%), and the Indian Journal of Orthopaedics (7 articles, 4.7%) (Table 4). To help authors easily and precisely submit their manuscripts, a visualization of cluster density was used to analyze the co-cited journals. As shown in Figure 4, these articles can be classified into three groups. The most cited blue cluster journals primarily published articles on joint surgery, while the green cluster journals often explored articles focused on fracture, and the red cluster journals were mainly interested in articles related to biological materials (Figure 4). Readers can retrieve and read periodicals from this range, which helps them improve their knowledge in this field.

**TABLE 4:** Top 5 productive journals in the field of additive manufacturing technology in pelvic surgery.

Rank	Journal	Number	Percentage
1	international orthopaedics	15	10.1
2	journal of orthopaedic surgery and research	10	6.8
3	orthopaedic surgery	9	6.1
4	bmc musculoskeletal disorders	8	5.4
5	indian journal of orthopaedics	7	4.7

### Analysis of authors

**FIGURE 5:**
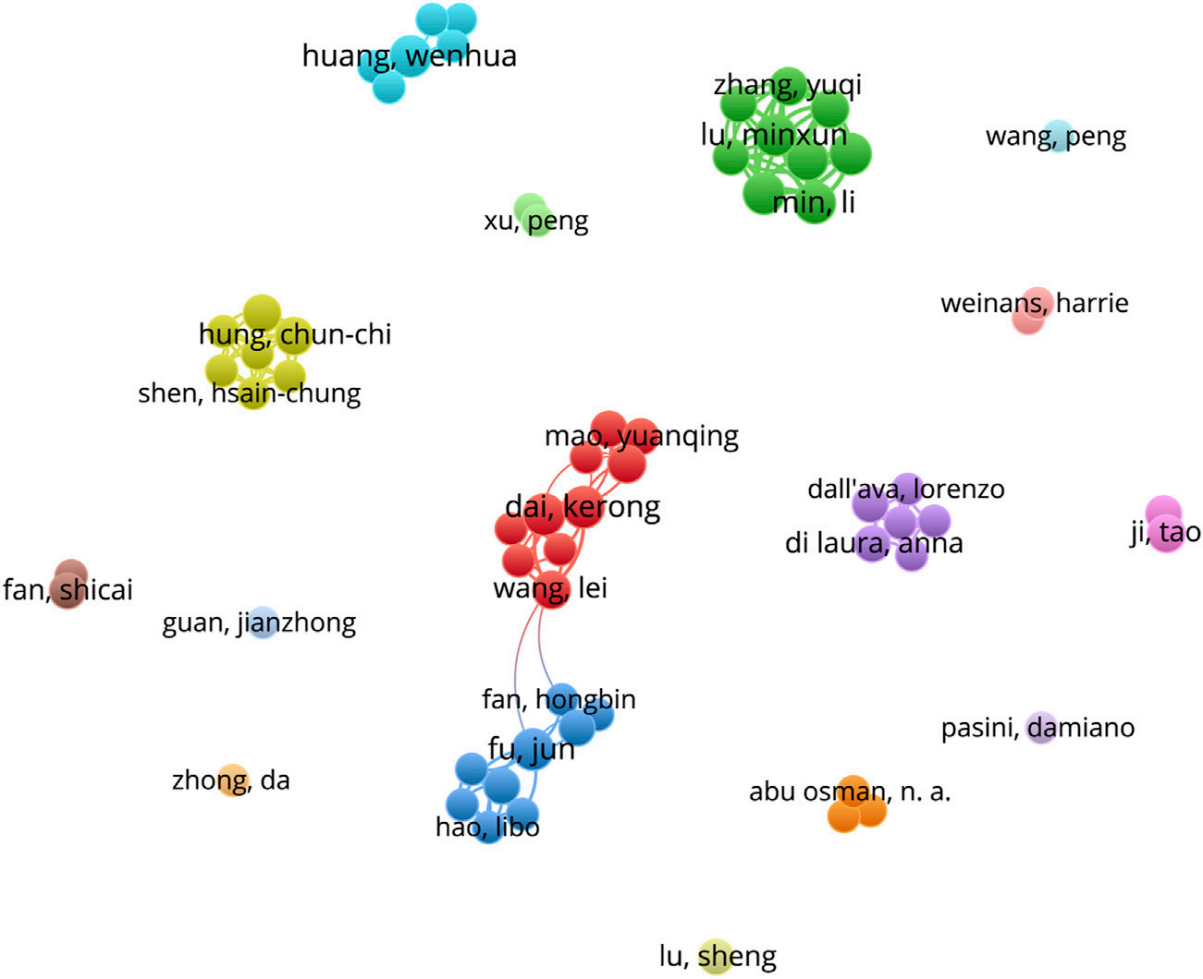
Cooperation network of productive authors. (The map’s bubbles represent authors, and the lines connecting the bubbles represent collaborative relationships. The larger the bubble area, the greater the number of publications.).

1,506 authors contributed to the selected 291 publications. The top 10 high-yield authors are listed in Table 5. T. Ji (139 citations) is the most cited of the aforementioned authors, while L. Wang (17 citations) is the least cited. The majority of the high-yield authors in this field are from Shanghai Jiao Tong University School of Medicine, and all the authors are Chinese. The network of author collaborations is shown in Figure 5. The lines between the bubbles on the network visualization signify collaboration relationships, while the authors are represented by the bubbles, whose areas indicate the number of published papers.

**TABLE 5:** The 10 high-yield authors in the field of additive manufacturing technology in pelvic surgery.

Rank	Author	Publications	Percentage	Affiliation
1	M.X. Lu	6	2.1	Sichuan University, China
2	K.R. Dai	6	2.1	Shanghai Jiao Tong University School of Medicine, China
3	Y.Q. Hao	6	2.1	Shanghai Jiao Tong University School of Medicine, China
4	J. Fu	6	2.1	Sun Yat-sen University, China
5	W.H. Huang	6	2.1	Southern Medical University, China
6	J. Wang	5	1.7	Southeast University, China
7	L. Wang	5	1.7	The Affiliated People’s Hospital of Jiangsu University, China
8	C.C. Hung	5	1.7	Tri-Service General Hospital and National Defense Medical Center, China
9	H.W. Li	5	1.7	Shanghai Jiao Tong University School of Medicine, China
10	T. Ji	5	1.7	Beijing Jishuitan Hospital, China

### Analysis of keywords

**FIGURE 6:**
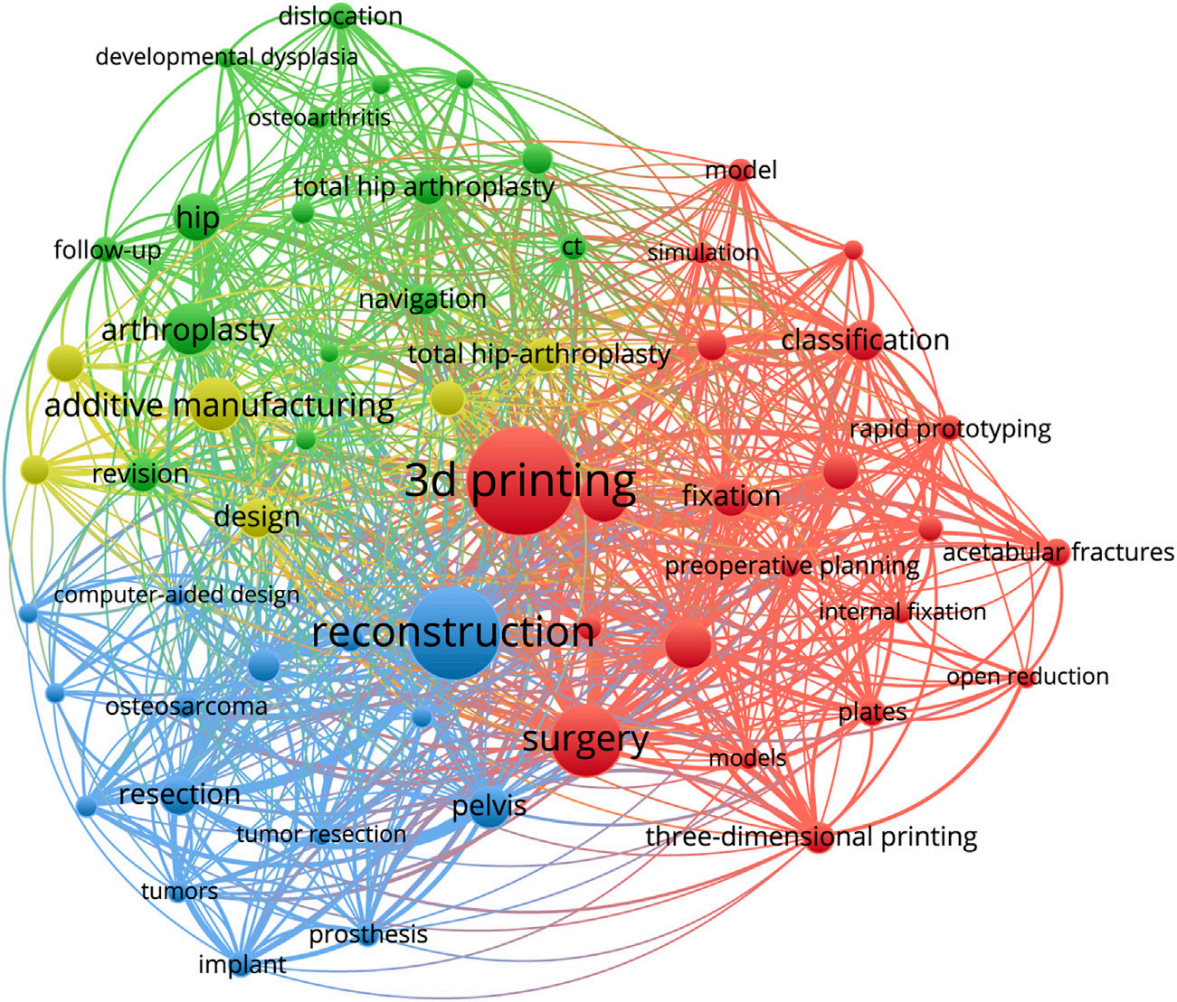
Network visualization of keywords. (The larger the bubbles are, the higher the probability of the keyword appearing. Then the more connections there are between the two bubbles, the more publications they appear in together, and the stronger the correlation will become. Furthermore, the different colors of the bubbles represent different clusters, implying that the keyword represented by the same color of bubble has a stronger correlation.).

Keywords summarized the main points of a research. It is possible to describe the research hotspots and trends in this field using keyword co-occurrence analysis. As illustrated in Figure 6, high-frequency keywords such as 3D printing, reconstruction, surgery, additive manufacturing, arthroplasty, and so on constitute this field’s research topic. Furthermore, the network visualization of keyword co-occurrence analysis revealed that the keywords are mainly divided into 3 clusters. Cluster 1 focuses on pelvic fractures, and its keywords include 3D printing, surgery, fixation, acetabular fractures, and so on; Cluster 2 focuses on total hip arthroplasty, and its keywords include additive manufacturing, arthroplasty, hip, total hip arthroplasty, and so on; Cluster 3 focuses on pelvic tumors, with the keywords reconstruction, pelvic, resection, tumors, implant, and so on. According to the findings, the use of additive manufacturing technology in pelvic surgery is primarily focused on pelvic fractures, pelvic tumors, and hip diseases.

## Discussion

The current study presents the findings of a bibliometric analysis of 291 articles on additive manufacturing technology in pelvic surgery conducted between January 2009 and November 2022 using the WoSCC database and VOSviewer software. The volume of publications shows that the development path from 2009 to the present is divided into two phases: 2009–2015, the early phase of slow development, and 2016-present, the era of rapid development. Furthermore, the year 2021 saw the most single-year publications in the field of additive manufacturing technology in pelvic surgery until November 2022. The overall trends suggest that more scholars will be engaged in this area in the future, contributing new scientific discoveries.

The analysis of countries, institutions, and authors enables the investigation of collaboration between various research topics. Based on the number of publications and average citations, China is the most productive and influential country for additive manufacturing technology in pelvic surgery. It is noteworthy that the top 5 most productive institutions and the top 10 authors are all from China. Moreover, the top 10 authors, K.R. Dai (Rank 2), Y.Q. Hao (Rank 3), and H.W. Li (Rank 9) are all from Shanghai Jiao Tong University School of Medicine. According to the three authors’ article (Hao et al., 2021; Wu et al., 2021; Kong et al., 2022), they have more and better collaboration, and they mostly publish articles on additive manufacturing technology in pelvic tumors, pelvic implants, pelvic fractures, and the disease of the hip joint. These results offer a very great prospect for additive manufacturing technology in pelvic surgery. Of course, the analysis of the above information reveals that the development of additive manufacturing technology in the field of pelvic surgery may be unbalanced, as the current state of research, primarily by Chinese scholars, may bring some geographical differences, implying that the study’s results may not be applicable to all humans. Furthermore, the visualization map shows weak connections, indicating a lack of coordination among countries, organizations, and authors. Therefore, global academic collaboration between countries/regions and institutions needs to be expanded.

In terms of journal information, we discovered that International Orthopaedic is the most productive journal in the field of additive manufacturing technology in pelvis surgery, as well as having the better results (Rank 4) in co-cited journals. Journal analysis can assist researchers in locating relevant publications for which to search and submit manuscripts. Keywords represent not only current research hotspots, but also future research trends. Through visual analysis of keywords, we identified the research hotspots in the field since the last decade. The most common keywords in the field of additive manufacturing technology in the pelvis surgery are “3D printing”, “reconstruction”, “surgery”, “additive manufacturing”, “arthroplasty”, “accuracy”, “hip”, “pelvis”, “fixation”, “resection”, “acetabular fracture”. In order to better grasp the current research hotspots, we thoroughly read and evaluated all retrieved articles and selected the top 10 most cited publications (Arabnejad Khanoki and Pasini, 2012; Cartiaux et al., 2014; Wong et al., 2015; Wu et al., 2015; Zeng et al., 2016; Arabnejad et al., 2017; Kim et al., 2017; Liang et al., 2017; Upex et al., 2017; Wei et al., 2017), carefully evaluating them in terms of Title, Purpose, Results, Limitations, Journal, and Citation (Table 6). According to current research, the application of additive manufacturing technology in pelvic surgery primarily addresses the following diseases: pelvic tumor, pelvic fracture, and hip joint disease (mainly total hip arthroplasty).

**TABLE 6:** Top 10 cited articles in the field of additive manufacturing technology in pelvic surgery.

Study	Title	Purpose	Results	Limitations	Journal	Citation
Arabnejad et al. (2017)	Fully Porous 3D Printed Titanium Femoral Stem to Reduce Stress-Shielding Following Total Hip Arthroplasty	Tuning material architecture of Total Hip Arthroplasty to achieve a substantial reduction of bone resorption secondary to stress shielding	A high strength fully porous material with tunable mechanical properties is introduced for use in hip replacement design	The study is still in the preliminary stages of research	Journal of Orthopaedic Research	203
Wong et al. (2015)	One-step reconstruction with a 3D-printed, biomechanically evaluated custom implant after complex pelvic tumor resection	Describing a novel workflow of performing a partial acetabular resection in a patient with pelvic chondrosarcoma and reconstruction with a custom pelvic implant in a one-step operation	The patient could walk unaided with a good hip function. No tumor recurrence and implant loosening were noted at 11 months after surgery	Further study in a larger population is needed to assess the clinical efficacy of the workflow in complex bone tumor surgery	Computer Aided Surgery	125
Arabnejad Khanoki and Pasini, (2012)	Multiscale design and multiobjective optimization of orthopedic hip implants with functionally graded cellular material	To solve the problems of bone-implant interface instability and bone resorption during total hip rarthroplasty	In this paper proposing a novel type of implant that can minimize concurrently bone résorption and implant interface failure	There are 5 optimizations available to improve this design	Journal of Biomechanical Engineering	107
Liang et al. (2017)	Reconstruction with 3D-printed pelvic endoprostheses after resection of a pelvic tumour	To report the feasibility of using 3D-printing technology for patients with a pelvic tumour who underwent reconstruction	The use of 3D-printed pelvic prostheses for reconstruction of the bony defect after resection of a pelvic tumour was safe, without additional complications, and gave good short-term functional results	The main limitation of this study is the relatively short follow-up	Bone & Joint Journal	100
Zeng et al. (2016)	A combination of three-dimensional printing and computer-assisted virtual surgical procedure for preoperative planning of acetabular fracture reduction	To evaluate the feasibility, accuracy and effectiveness of performing 3D printing technology and computer-assisted virtual surgical procedures for preoperative planning in acetabular fractures	The 3D printing technology combined with virtual surgery for acetabular fractures is feasible, accurate, and effective leading to improved patient-specific preoperative planning and outcome of real surgery	long-term outcomes are not known, and need to be followed up	Injury	88
Cartiaux et al. (2014)	Improved Accuracy with 3D Planning and Patient-Specific Instruments During Simulated Pelvic Bone Tumor Surgery	The study investigated accuracy of patient-specific instru-mentation (PSI) for bone-cutting during simulated tumor surgery within the pelvis	Using PSI technology during simulated bone cuts of the pelvis can provide good cutting accuracy	The strudy does not exactly represent actual pelvic surgery	Annals of Biomedical Engineering	84
Wu et al. (2015)	Printed Three dimensional Anatomic Templates for Virtual Preoperative Planning Before Reconstruction of Old Pelvic Injuries: Initial Results	To assess the use of three-dimensional (3D) printing techniques for surgical management of old pelvic fractures	Preoperative planning using the 3D printed models was feasible in all cases	the small number of patients and the absence of long-term follow-up data	Chinese Medical Journal	66
Upex et al. (2017)	Application of 3D printing for treating fractures of both columns of the acetabulum: Benefit of pre-contouring plates on the mirrored healthy pelvis	The purpose of this technical note is to describe how we used 3D printing as an aid to treat acetabular fractures	3D printing is a highly relevant and an easy-to-use technology from a clinical point of view for treating complex fractures	For now, the limiting factor is the time needed to print a full-size (1:1 model) of the hemipelvis	Orthopaedics & Traumatology: Surgery & Research	65
Wei et al. (2017)	One-step reconstruction with a 3D-printed, custom-made prosthesis after total *en bloc* sacrectomy: a technical note	To describe the design of a 3D-printed custom-made prosthesis for reconstruction after total *en bloc* sacrectomy, the surgical technique, and the clinical and functional outcome of a patient	The patient received one-stage total *en bloc* sacrectomy through posterior approach followed by reconstruction with the 3D-printed sacral prosthesis	The report is of only one case	European Spine Journal	63
Kim et al. (2017)	Sacral Reconstruction with a 3D-Printed Implant after Hemisacrectomy in a Patient with Sacral Osteosarcoma: 1-Year Follow-Up Result	Hemisacrectomy and sacral reconstruction using a 3D-printed implant	This is the first report of a case of hemisacral recon-struction using a custom-made 3D-printed implant	Distinct from conventional implants, the strength of 3D-printed im-plants may not be guaranteed	Yonsei Medical Journal	57

## Pelvic tumor

The third most typical site for metastatic bone cancers is the pelvis, which frequently displays pain and limited movement clinically (Han et al., 2019). Tumor resection and pelvic reconstruction are common steps in pelvic tumor surgery. Due to the irregularity of pelvic anatomy, the proximity of important blood vessels and nerves, and the limited surgical access, pelvic tumor surgery is significantly more challenging. The success of pelvic tumor surgery depends on the complete removal of the tumor and a successful pelvic reconstruction. Additive manufacturing technology has demonstrated unparalleled superiority in pelvic tumor surgery due to its personalization, high-precision, and rapidly accessible manufacturing (Chen et al., 2016). Preoperative surgical planning, intraoperative guides for precise osteotomy planes, and implant reconstruction with 3D printed prostheses have led to the increasing application of additive manufacturing technology in pelvic tumor surgery (Biscaccianti et al., 2022).

A 3D printing workflow for pelvic tumor surgery was described by Wong et al. (2015). The full scope of the tumor was firstly outlined, and its volume was extracted from computed tomography (CT) or magnetic resonance imaging (MRI) images, followed by the creation of a 3D bone tumor model and the design of tumor resection margins. Then, the shape of the virtual resected bone defect was reconstructed by overlapping mirrors in the unaffected relative pelvic region, and a patient-specific pelvic implant was designed while taking surgical access and surrounding soft tissues into account. After that, to complete the biomechanical evaluation, the designed implant is subjected to finite element analysis using dedicated engineering software. During the procedure, the tumor was removed using a Patient-specific instrument (PSI) according to a predetermined plan, and the bone defect area was well adapted with a patient-specific computer-aided design (CAD) implant, which was subsequently fixed. The results of this study also imply that tumor PSI may facilitate more challenging multiplanar osteotomies in the complex anatomical pelvic region. This enables the surgeon to remove the bone tumor with sufficient margins and to preserve the most normal bone possible for improved functional restoration. To investigate the accuracy of patient-specific instrument (PSI) for bone cutting during pelvic tumor simulation, 24 surgeons (10 senior and 14 junior) were asked to perform tumor resection, and the results revealed no significant differences in terms of the position of the cutting plane and the surgical margins obtained between senior and junior surgeons (Cartiaux et al., 2014).

A study found that using 3D-printed pelvic prostheses to reconstruct defects after pelvic tumor resection is feasible and safe, with good short-term functional outcomes (Liang et al., 2017). When it comes to the long-term outcomes of reconstructive surgery, rigid fusion between bone and implant has been a major issue. 3D-printed prostheses, as compared to other implants, can be contoured to fit the bone defect more precisely and machined with a porous bone contact surface to guide bone growth and improve long-term stability (Shah et al., 2016). The results of a 1-year follow-up of sacral reconstruction with 3D-printed implants after hemisacrectomy in patients with sacral osteosarcoma show that good bone connections can be achieved on both densely structured strut surfaces and loosely structured porous meshes (Kim et al., 2017). All the evidence suggests that additive manufacturing technology is already playing an important role in the field of pelvic oncology surgery and will become even more indispensable in the future.

## Pelvic fracture

Pelvic fractures are a common type of fracture in clinical practice, mostly caused by high-energy injuries such as traffic accidents, industrial accidents, and falls from height. Complex pelvic and acetabular anatomy makes clinical diagnosis and therapy rather challenging. Researchers have never stopped looking into pelvic fractures since they are frequently accompanied by severe shock and pelvic organ injury, which has a high mortality and disability rate (Tachibana et al., 2009). The major objectives of pelvic fracture surgery are to restore the symmetry and integrity of the pelvic ring, pay attention to handling soft tissues carefully, hasten postoperative healing and early rehabilitation, and improve long-term hip joint function (Halvorson et al., 2014). Additive manufacturing technology, which is widely used in preoperative diagnosis and planning of pelvic fractures, intraoperative navigation, and prosthetic endoprostheses, among other things, can assist clinicians in diagnosis and treatment by realizing a panoramic simulation of pelvic structures. The first polyethylene pelvic model was reportedly used in orthopedic surgery in 1979, with excellent results (Tonner and Engelbrecht, 1979).

There are several aspects to the use of additive manufacturing technology in the surgical treatment of pelvic fractures. To start, it can print a 1:1 3D model to assist orthopedic surgeons in understanding the extent and type of fracture as well as the displacement of the fracture mass. This will enable them to make a precise preoperative diagnosis, weigh potential intraoperative risks, and develop the best surgical plan. Second, additive manufacturing technology can “tailor” personalized implants to match the patient’s bones more precisely when used in conjunction with intraoperative navigation technology, effectively shortening the operation time, significantly reducing the incidence of postoperative infections and other complications, and restoring function of the affected limb more quickly. Furthermore, the 3D-printed navigation template aids in determining the precise location of the bone plate and screws, and the combination of the navigation template and pre-curved fixation can accurately and effectively repair the pelvic fracture (Chen et al., 2017). Cimerman and Kristan (2007) described a surgical planning software for pelvic and acetabular fractures with a mouse-based CAD-style interface that produced personalized, precise surgical plans that greatly increased the success rate of surgery. And in other studies, 3D printed models applied to pelvic fracture surgery significantly reduced operative time, surgeon fatigue, and blood loss (Starosolski et al., 2014).

## Hip joint disease

With the aging trend, the number of patients requiring total hip arthroplasty grows each year. Total hip arthroplasty (THA) has emerged as one of the most effective treatments for advanced hip disease (femoral head necrosis, hip dysplasia, femoral neck fracture, degenerative hip arthritis, rheumatoid arthritis, ankylosing spondylitis, etc.). For patients with hip dysplasia and total hip revision, the emergence of additive manufacturing technology undoubtedly opens a whole new path of personalized and precise medical treatment.

Hip dysplasia is typically imaged by showing diminished acetabular coverage of the femoral head, a shallow and thin acetabular wall, acetabular bone abnormalities, and osteosclerosis (Du et al., 2020). Clinical management of severe hip dysplasia is frequently difficult due to the severity of the acetabular defect and significant femoral head dislocation. Using additive manufacturing technology to design a 1:1 pelvic model, which can clearly demonstrate the anatomical morphology of the patient’s hip joint and help the surgeon understand the true and false socket positions in relation to the acetabular wall defect condition. In turn, it can successfully benefit the surgeon by enhancing preoperative planning, obtaining the accurate hip joint’s center of rotation, and enhancing surgical accuracy and safety (Won et al., 2013). An personalized titanium tricompartmental cup, first proposed by Christie et al. (2001) is a custom-made acetabular prosthesis with three winged projections attached to the outer edge of the cup that can be screwed to the ilium, pubic bone, and sciatic bone, respectively, when the acetabular defect is very large. It allows for the precise and stable restoration of acetabular anatomy, as well as the restoration of strong bone contact and hip biomechanics. However, it is crucial to note that fitting a custom triple wing socket cup necessitates significant iliac bone exposure, which increases the risk of nerve and vascular injury (Christie et al., 2001).

Furthermore, additive manufacturing technology is becoming more widely used in total hip revision. Despite technological advancements that have resulted in the success of current total hip arthroplasties, more than 13% of hip prostheses still require revision surgery due to bone resorption and aseptic implant loosening (Kurtz et al., 2007). In 2017, Hughes et al. conducted a study of 3D printed pelvic models in hip revision patients, using a pelvic model for surgical rehearsal prior to revision surgery, grinding layer by layer and anticipating the size of the socket cup. Depending on the size of the bone defect, structural bone grafting or tantalum metal pads were used for simulation, with screw positions and trajectories determined to ensure prosthetic stability while avoiding screw damage to blood vessels or nerves (Hughes et al., 2017). As a result, there was better preoperative planning, less operative time, and excellent outcomes. Further research has shown promising early results in hip revision using 3D printed titanium trabecular lattice structures to fill and reconstruct extensive acetabular bone defects (Colen et al., 2013). It is worth noting that Arabnejad et al. (2017) worked on new fully porous 3D printed titanium femoral stems to reduce stress shielding after total hip arthroplasty and to lower the incidence of total hip revision.

The field of medical education has also been revolutionized by additive manufacturing technology today. For many years, the teaching methods for medical students and physicians have remained at the level of flat images. Traditional teaching methods have significant drawbacks for orthopedic clinical education with complicated spatial structure and demanding a lot of spatial concept capacity. As a result of the development of additive manufacturing technology, two-dimensional elements are transformed into three-dimensional conformations and presented as interactive software or printed models, enabling students to fully comprehend the spatial structure of real objects without the use of any material objects (Abdulcadir et al., 2020). Taking into account the complicated anatomy of the pelvis, 3D printed models can guide newcomers in understanding the borders of pelvic tumors, the typology of complex pelvic fractures, etc (Lee et al., 2022; Wu et al., 2022). Moreover, physicians can also quickly develop their operating skills while learning *via* surgical training based on 3D printed models. Overall, additive manufacturing technology is regarded as the best educational tool among the ones that are now on the field.

In general, the outcomes of additive manufacturing technology in pelvic surgery are promising. However, its application in this field has some limitations. First, some researchers have revealed that creating 3D models typically requires a lot of time, rendering them unsuitable for application in emergency medical circumstances (Lopez et al., 2021). Second, no 3D printed product is capable of fully replicating and displaying bone, muscle, skin, and other tissues. Third, the cost of establishing 3D printing facilities (hardware and software) is prohibitively expensive for most of the medical institutions, and as a result, the treatment fee is also expensive for patients. Meanwhile, in this study, as with prior bibliometric assessments, it is inevitable that it will be difficult to fully collect all the literature related to this field in the WoSCC database due to the differences in literature search algorithms and the speed of literature updates. Nevertheless, this study can still be used to illustrate the general states and trends of research topics.

## Conclusion

In this study, bibliometric analysis was employed for comprehensively and systematically reviewing of publications related to additive manufacturing technology in pelvic surgery. In addition, the relevant research results were presented and discussed in detail using visualization tools, revealing that 3D printing technology is the hottest field of pelvic surgery and will continue to play an important role in the future of pelvic surgery.

## Data Availability

The original contributions presented in the study are included in the article/Supplementary Material, further inquiries can be directed to the corresponding author
